# Tophus goutteux de la main révélant une goutte

**DOI:** 10.11604/pamj.2014.17.165.3910

**Published:** 2014-03-06

**Authors:** Monsef Boufettal, Mohamed Azouz

**Affiliations:** 1 Orthopedic Surgery Department of Ibn Sina hospital, Rabat, Morocco

**Keywords:** Tophus goutteux, goutte, tophus, gouty tophi, gout, tophi

## Image en medicine

Les tophi goutteux sont des nodules indolores sous-cutanés de taille très variable, parfois réunis entre eux en une masse bosselée, de localisations diverses et pouvant révéler une goutte d'évolution chronique. Nous rapportons un cas de tophus goutteux de la face dorsale de la main révélant une goutte. Il s'agit d'un patient de 67 ans, insuffisant rénal chronique qui présente depuis 2 ans une tuméfaction de la face dorsale de la main droite en regard des articulations métacarpo-phalangiennes du troisième et quatrième rayon qui augmentait progressivement de volume sans déficit sensitivomoteur et sans aucun autre symptôme. L'examen clinique trouve une masse mesurant 3cm de grand axe, dur, mobile par rapport au plan superficiel et profond et non douloureuse. La mobilité des articulations métacarpo-phalangiennes du troisième et quatrième doigt est respectée sans déficit d'extension ni de flexion. La radiographie avait montré des signes d'arthropathie destructrice et le bilan biologique avait objectivé une hyperurécemie à 512 µmol/l. le diagnostic d'un tophus goutteux a été retenu et le patient a été mis sous traitement par la colchicine avec une bonne évolution clinique. Le tophus est la lésion pathognomique de la goutte. C'est un dépôt d'urates, sous forme cristalline ou amorphe, entouré d'une réaction inflammatoire. Les tophi sont localisés le plus souvent dans le pavillon de l'oreille, le coude, le gros orteil, le talon, le dos du pied, le tendon d'Achille et plus rarement la main, et sont dans plusieurs cas révélateurs d'une goutte le plus souvent chronique.

**Figure 1:**
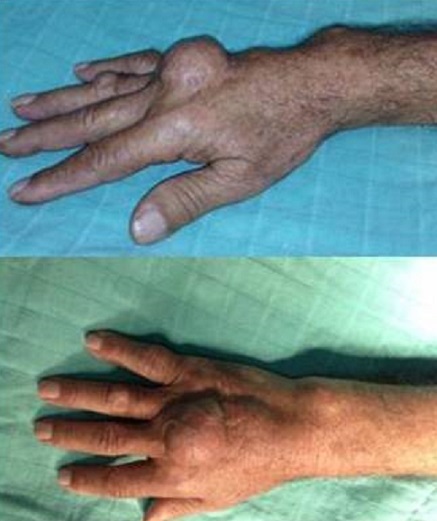
Tophus goutteux de la face dorsale de la main droite

